# Machine learning-based prediction models for noninvasive respiratory support failure in acute respiratory failure: a systematic review and meta-analysis

**DOI:** 10.3389/fmed.2026.1775670

**Published:** 2026-04-10

**Authors:** Kadir Burak Akgün, Hajed M. Al-Otaibi, Gaspar R. Chiappa, Abdullah A. Alqarni, David Troxell, Franciszek Przybylski, Antonio M. Esquinas

**Affiliations:** 1 Department of Pulmonology, Faculty of Medicine, Hatay Mustafa Kemal University, Hatay, Türkiye; 2 Department of Respiratory Therapy, Faculty of Medical Rehabilitation Sciences, King Abdulaziz University, Jeddah, Saudi Arabia; 3 Faculty of Health Sciences, Universidad Autónoma de Chile, Providencia, Santiago, Chile; 4 Graduate Program, Eastern University, Philadelphia, PA, United States; 5 Student Scientific Society at 2nd Department of Pulmonary Diseases, Lung Cancer and Internal Medicine, Medical University of Białystok, Białystok, Poland; 6 Intensive Care Unit, Hospital Meseguer Meseguer.NIV-ICU Group, Biomedical Research Institute Pascual Parrilla–IMIB, Murcia, Spain

**Keywords:** acute respiratory failure, artificial intelligence, high flow nasal cannula, machine learning, noninvasive ventilation

## Abstract

**Background:**

Early identification of noninvasive respiratory support (NIRS) failure in acute respiratory failure (ARF) is clinically relevant, as delayed intubation is associated with worse outcomes. Machine learning-based prediction models have been proposed to support escalation decisions, but their performance and reliability remain uncertain.

**Objective:**

To systematically evaluate the discriminative performance of machine learning-based models for predicting NIRS failure in adults with ARF.

**Methods:**

We conducted a systematic review and meta-analysis following PRISMA 2020 guidelines and registered the protocol in PROSPERO (CRD420251167330). PubMed, Web of Science, and Scopus were searched from January 2010 to the final search date. Cohort studies developing or validating machine learning models to predict NIRS failure, primarily defined as endotracheal intubation, were included. Discrimination was assessed using the area under the receiver operating characteristic curve (AUC). Logit-transformed AUCs were synthesized using random-effects models with restricted maximum likelihood estimation and Hartung–Knapp confidence intervals. Risk of bias and certainty of evidence were assessed using PROBAST-AI and GRADE, respectively.

**Results:**

Fourteen cohort studies comprising 34,500 patients were included. The descriptive pooled AUC was 0.84 (95% CI, 0.78–0.89) with extreme heterogeneity (I^2^ = 99.5%) and wide prediction intervals. Subgroup analyses showed no statistically significant differences by validation strategy or type of noninvasive respiratory support. All studies were rated at high risk of bias, and the certainty of evidence was very low.

**Conclusion:**

Machine learning-based models demonstrate moderate discrimination; however, extreme heterogeneity, high risk of bias, and very low certainty of evidence preclude clinical implementation.

**Systematic review registration:**

https://www.crd.york.ac.uk/PROSPERO/view/CRD420251167330.

## 1 Introduction

Noninvasive respiratory support strategies, including noninvasive ventilation (NIV) and high-flow nasal cannula (HFNC), are widely used in the management of acute respiratory failure (ARF) to improve gas exchange while avoiding complications associated with invasive mechanical ventilation, such as ventilator-induced lung injury, ventilator-associated pneumonia, and hemodynamic instability (1–6). When appropriately applied, these modalities can reduce intubation rates and improve clinical outcomes; however, failure of noninvasive respiratory support remains common and is strongly associated with increased morbidity and mortality, particularly when escalation to invasive ventilation is delayed (7, 8).

Clinical tools such as the HACOR score and the ROX index have been developed to assist clinicians in identifying patients at risk of NIV or HFNC failure and to guide timely decisions regarding intubation (9–11). Despite their clinical utility, these scores rely on a limited number of static physiological variables and demonstrate inconsistent performance across heterogeneous ARF populations. Moreover, the absence of universally accepted thresholds and the continued reliance on clinician judgment highlight the inherent uncertainty surrounding early prediction of noninvasive respiratory support failure in routine practice.

Recent advances in machine learning and artificial intelligence have led to the development of predictive models capable of integrating high-dimensional and dynamic clinical data to estimate the risk of noninvasive respiratory support failure (12, 13). Although individual studies have reported promising discriminative performance, substantial variability exists in model design, validation strategies, outcome definitions, and reporting quality. The extent to which these models provide reliable, generalizable, and clinically meaningful predictions remains unclear (14, 15). We therefore conducted a systematic review and meta-analysis to critically evaluate the predictive performance, risk of bias, and certainty of evidence of machine learning–based models for predicting failure of noninvasive respiratory support in adults with acute respiratory failure.

## 2 Materials and methods

### 2.1 Study design and protocol registration

We conducted a systematic review and meta-analysis of prediction model studies in accordance with the Preferred Reporting Items for Systematic Reviews and Meta-Analyses (PRISMA) 2020 statement (Supplementary material 1). The review protocol was prospectively registered in PROSPERO (CRD420251167330). Given the focus on prediction models, the methodological approach was additionally informed by principles underlying TRIPOD and PROBAST-AI guidance for prognostic model evaluation.

### 2.2 Population

We included studies enrolling adults (≥18 years) hospitalized with acute respiratory failure of any etiology who were managed with noninvasive respiratory support, including noninvasive ventilation (NIV) and/or high-flow nasal cannula (HFNC).

### 2.3 Prediction models

Eligible studies developed, internally validated, or externally validated machine learning–based prediction models intended to estimate the risk of noninvasive respiratory support failure. Traditional clinical scores without a machine learning component were excluded.

### 2.4 Outcome

The primary outcome was failure of noninvasive respiratory support, defined as the need for endotracheal intubation or initiation of invasive mechanical ventilation. Mortality and composite outcomes incorporating intubation and death were recorded descriptively but were not pooled quantitatively.

### 2.5 Study designs

We included prospective and retrospective cohort studies reporting model discrimination performance. Randomized controlled trials, case reports, reviews, editorials, conference abstracts without full text, pediatric studies, and studies unrelated to acute respiratory failure were excluded.

### 2.6 Information sources and search strategy

We systematically searched PubMed, Web of Science, and Scopus from 1 January 2010 to 17 October 2025. The search strategy combined controlled vocabulary and free-text terms related to acute respiratory failure, noninvasive respiratory support, machine learning, artificial intelligence, and prediction models. Reference lists of included studies were manually screened to identify additional eligible articles. Only studies published in English were included. The full search strategy for each database is provided in the Supplementary material.

### 2.7 Study selection

After removal of duplicate records, studies retrieved were entered into Rayyan software,^1^ where two reviewers independently screened titles and abstracts for eligibility. Full texts of potentially relevant articles were assessed independently using predefined inclusion and exclusion criteria. Discrepancies were resolved by consensus or consultation with a third reviewer. The study selection process is presented in a PRISMA flow diagram.

### 2.8 Data extraction

Two reviewers independently extracted data using a standardized form. Extracted variables included study characteristics, population features, type of noninvasive respiratory support, machine learning algorithms, validation strategy (internal or external), outcome definition, and reported model performance metrics.

To minimize optimism bias, performance estimates were extracted preferentially from external validation datasets when available. When external validation was not performed, internally validated results were used. When multiple models were reported within a single study, we extracted performance metrics for the model pre-specified by the authors as the primary model, while acknowledging the potential for overestimation inherent to this approach.

### 2.9 Risk of bias and applicability assessment

We assessed risk of bias and applicability using the Prediction Model Risk of Bias Assessment Tool for Artificial Intelligence (PROBAST-AI). Two reviewers independently evaluated each study across the domains of participants, predictors, outcome, and analysis. Overall judgments of risk of bias and applicability were classified as low, high, or unclear. Disagreements were resolved through discussion or third-party adjudication.

### 2.10 Statistical analysis

We synthesized model discrimination using the area under the receiver operating characteristic curve (AUC). Because AUC is bounded (0 to 1), we pooled logit-transformed AUC estimates and back-transformed summary effects for reporting. When studies did not report uncertainty measures, we derived standard errors using established approximations based on sample size and event counts, and we prioritized estimates from external validation datasets when available. We performed random-effects meta-analysis using restricted maximum likelihood (REML) to estimate between-study variance, and we computed confidence intervals using the Hartung–Knapp adjustment. We quantified heterogeneity using *I*^2^ and *τ*^2^ and additionally reported prediction intervals to reflect the expected range of model performance in new settings. We prespecified subgroup analyses by validation strategy (external vs. internal) and by noninvasive respiratory support modality (NIV vs. HFNC vs. mixed) and treated these analyses as exploratory given residual heterogeneity. We assessed robustness through influence and sensitivity analyses, including leave-one-out analyses and exclusion of the largest and extreme-performance studies. We conducted all analyses and created all images in R (version 4.5.2).

### 2.11 Certainty of evidence

We evaluated the overall certainty of evidence for the primary outcome using the Grading of Recommendations, Assessment, Development, and Evaluation (GRADE) framework, adapted for prognostic research. Certainty was rated as high, moderate, low, or very low based on risk of bias, inconsistency, indirectness, imprecision, and publication bias.

## 3 Results

### 3.1 Study selection

**Figure 1:**
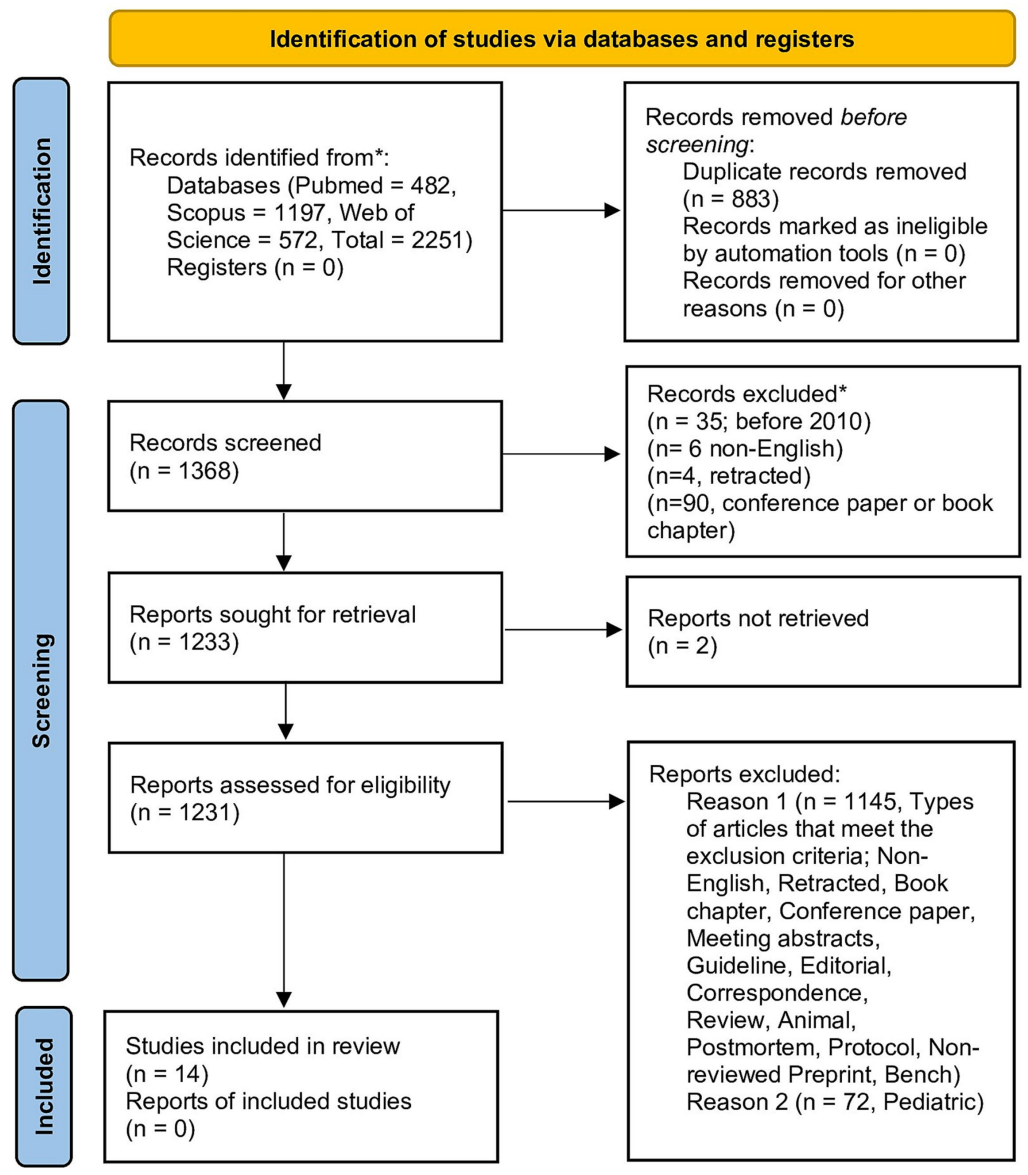
PRISMA flow diagram. PRISMA 2020 flow diagram illustrating the study selection process. Of 2,251 records identified through database searching, 14 studies met the predefined eligibility criteria and were included in the qualitative synthesis and quantitative analyses.

The systematic search yielded 2,251 records. After removal of 883 duplicates, 1,368 unique records were screened by title and abstract. Eighty-four articles underwent full-text assessment, of which three could not be retrieved despite repeated attempts. Fourteen studies met the predefined eligibility criteria and were included in the qualitative synthesis and quantitative analyses. The study selection process is illustrated in the PRISMA flow diagram (Figure 1).

### 3.2 Study characteristics

The included studies comprised a total of 62,221 adults in the qualitative synthesis. In accordance with the prespecified analytical hierarchy prioritizing external validation, 34,500 patients contributed to the quantitative meta-analysis. Clinical contexts varied substantially and included COVID-19–related acute hypoxemic respiratory failure, chronic obstructive pulmonary disease exacerbations, and mixed etiologies of ARF. Noninvasive ventilation (NIV), high-flow nasal cannula (HFNC), or mixed noninvasive strategies were evaluated.

Six studies reported external validation, whereas eight relied exclusively on internal validation. In accordance with the prespecified analytical hierarchy prioritizing generalizability, performance estimates derived from external validation cohorts were preferentially extracted when available. Consequently, 34,500 patients contributed to the quantitative synthesis. Study characteristics and extracted performance metrics are summarized in Table 1.

**Table 1:** Characteristics of the included studies.

Author	Year	Proposed AI model	Patient characteristic	Validation type	Failure criteria	NIRS type	Timing	*N* (Total)	*N* (for statistics)	AUC	Min CI	Max CI
Bendavid et al. (16)	2022	XGBoost	COVID-19	Internal + External	Intubation	Mixed	First 6–24 h	12,877	1,061	0.91	0.902	0.916
Liang et al. (17)	2022	SMSN	General ARF	Internal	Intubation	NIV	First 1–2 h	499	499	0.915	0.883	0.947
Essay et al. (18)	2022	LSTM	General ARF	Internal	Intubation	Mixed	First 12 h	22,075	22,075	0.9636	0.960	0.967
Cheng et al. (19)	2024	Ensembled	General ARF	Internal + External	Intubation	HFNC	No specific h	761	180	0.75	0.674	0.826
Carmichael et al. (20)	2021	XGBoost	COVID-19	Internal + External	Intubation	Mixed	First 48 h	14,470	259	0.65	0.546	0.754
Wang et al. (21)	2022	CatBoost	General ARF	Internal + External	Intubation,death	NIV	No specific h	1,348	419	0.846	0.82	0.92
Li et al. (23)	2025	SVE	General ARF	Internal	Intubation, tracheostomy	HFNC	First 4 h	427	427	0.839	0.786	0.889
Wang et al. (22)	2024	RF	General ARF	Internal	Intubation	HFNC	No specific h	700	700	0.831	0.826	0.836
Yang et al. (24)	2024	XGBoost	COVID-19	Internal	Intubation,death	HFNC	First 24 h	984	984	0.707	0.671	0.743
Nguyen et al. (25)	2023	Fusion	COVID-19	Internal	Intubation	Mixed	First 24 h	2,481	2,481	0.874	0.80	0.94
Odeyemi et al. (26)	2024	GBM	CAP	Internal	Intubation,death	Mixed	First 6 h	4,379	4,379	0.713	0.693	0.733
Douville et al. (27)	2021	RF	COVID-19	Internal	Intubation,death	Mixed	24 h-14 d	398	398	0.858	0.841	0.874
Liu et al. (28)	2024	Chat-GPT	General ARF	External	Intubation	HFNC	First 48 h	71	71	0.821	0.698	0.943
Yu et al. (12)	2025	SVM	General ARF	Internal + External	Intubation,death	HFNC	First 2 h	751	567	0.82	0.782	0.858

AI, artificial intelligence; ARF, acute respiratory failure; AUC, area under curve; CAP, community-acquired pneumonia; CI, confidence interval; d, days; GBM, gradient boosting machine; h, hours; HFNC, high-flow nasal cannula; LSTM, long short-term memory; NIRS, non-invasive respiratory support; NIV, non-invasive ventilation; RF, random forest; SMSN, stacking and modified SMOTE algorithm; SVE, soft voting ensemble.

### 3.3 Qualitative synthesis and outcome heterogeneity

Due to extreme heterogeneity and inconsistent reporting, a robust quantitative stratification for all sub-groups was not feasible. A quantitative stratification by time horizon (e.g., early vs. late failure) or specific etiology (e.g., chronic obstructive lung disease) was statistically infeasible due to highly variable follow-up periods—with some studies completely omitting timeframes—and a lack of undifferentiated ARF stratification in the primary texts. Outcome definitions were highly decision-dependent (ranging from early intubation to composite outcomes including mortality), preventing generalizability across different clinical workflows. Furthermore, evaluation of model implementability was severely limited: reporting of calibration metrics (such as calibration plots, slope/intercept, or Brier scores) and clinical utility assessments (e.g., Decision Curve Analysis) was largely absent across the included studies.

### 3.4 Overall discriminative performance

Across all studies, the descriptive pooled discriminative performance of machine learning–based models for predicting failure of noninvasive respiratory support yielded an AUC of 0.84 (95% CI, 0.78–0.89). Given the bounded nature of the AUC, estimates were synthesized using logit-transformed AUC values within a random-effects model with restricted maximum likelihood estimation, and confidence intervals were derived using the Hartung–Knapp method.

**Figure 2:**
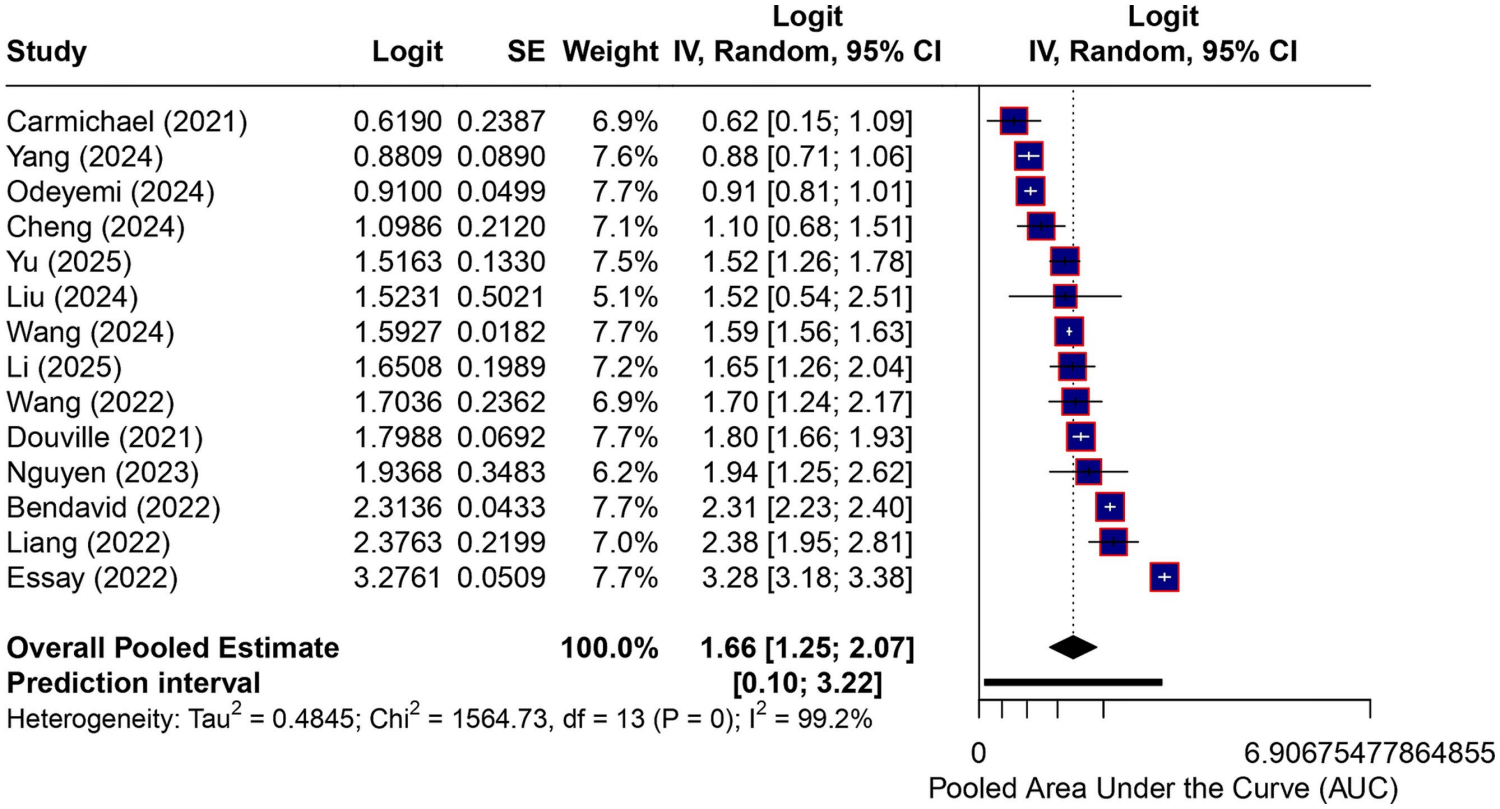
Overall discriminative performance of machine learning models. Forest plot showing the discriminative performance of machine learning–based prediction models for failure of noninvasive respiratory support in acute respiratory failure, expressed as the area under the receiver operating characteristic curve (AUC). Effect estimates are displayed on the logit (AUC) scale and were back-transformed to AUC values for interpretation. Summary estimates represent descriptive pooled discrimination derived from a random-effects model with restricted maximum likelihood estimation and Hartung–Knapp confidence intervals. Between-study heterogeneity was extreme (*I*^2^ = 99.5%). The pooled estimate should be interpreted as a descriptive summary rather than a precise or generalizable measure of predictive performance. The prediction interval reflects the expected range of model performance in future clinical settings.

Between-study heterogeneity was extreme (*I*^2^ = 99.5%), indicating substantial variability in reported model performance. As detailed in Table 1, the included cohorts exhibited diverse case-mixes, variations in NIRS modalities, and disparate definitions of failure. The corresponding prediction interval was wide, reflecting marked uncertainty in the expected discriminative performance of these models when applied to new clinical settings. Accordingly, the pooled AUC should be strictly as a descriptive summary of reported discrimination rather than as a precise or generalizable clinical benchmark (Figure 2).

### 3.5 Subgroup analyses

#### 3.5.1 Validation strategy

**Figure 3:**
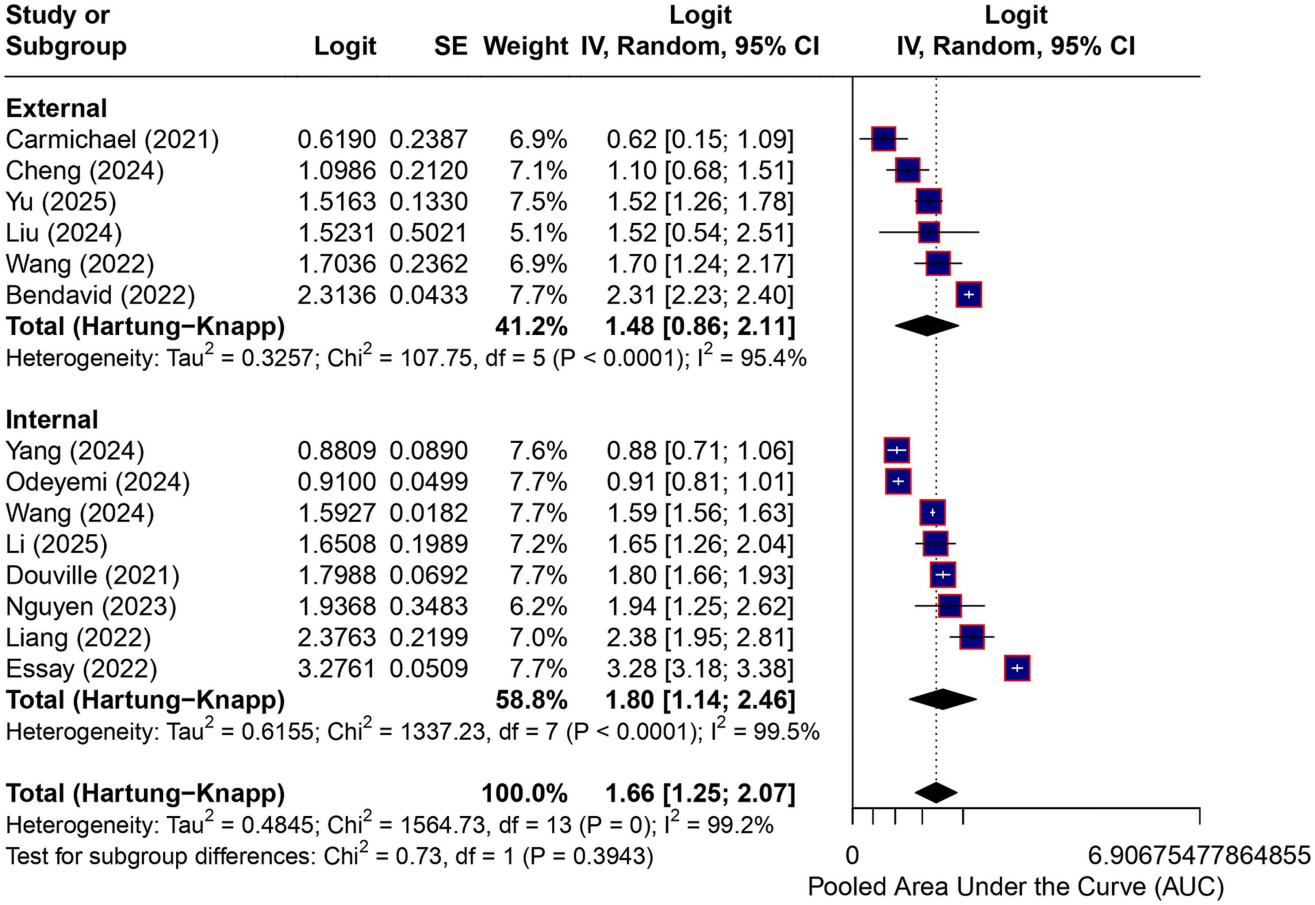
Subgroup analysis by validation strategy. Subgroup analysis of model discrimination stratified by validation strategy (external vs. internal validation). Effect estimates are displayed on the logit (AUC) scale and were back-transformed to AUC values for interpretation. Pooled estimates were obtained using random-effects models with restricted maximum likelihood estimation and Hartung–Knapp confidence intervals. Substantial heterogeneity persisted within both subgroups, with wide and overlapping prediction intervals. The test for subgroup differences was not statistically significant (*p* = 0.39), indicating insufficient evidence to support differential predictive performance by validation strategy. Subgroup analyses are exploratory and not intended for inferential comparisons.

In subgroup analyses stratified by validation strategy using logit-transformed AUCs and the Hartung–Knapp adjustment, external validation cohorts demonstrated a pooled AUC of 0.81 (95% CI, 0.70–0.89), while internal validation cohorts showed a pooled AUC of 0.86 (95% CI, 0.76–0.92). Heterogeneity remained substantial within both subgroups, and prediction intervals overlapped widely. The test for subgroup differences was not statistically significant (*p* = 0.39), indicating insufficient evidence to support differential discriminative performance between internally and externally validated models. Subgroup estimates overlapped substantially, and residual heterogeneity remained high. Consequently, these analyses do not allow inferences regarding model generalizability, and reported performance is likely to vary across clinical settings and populations (Figure 3).

### 3.6 Type of noninvasive respiratory support

**Figure 4:**
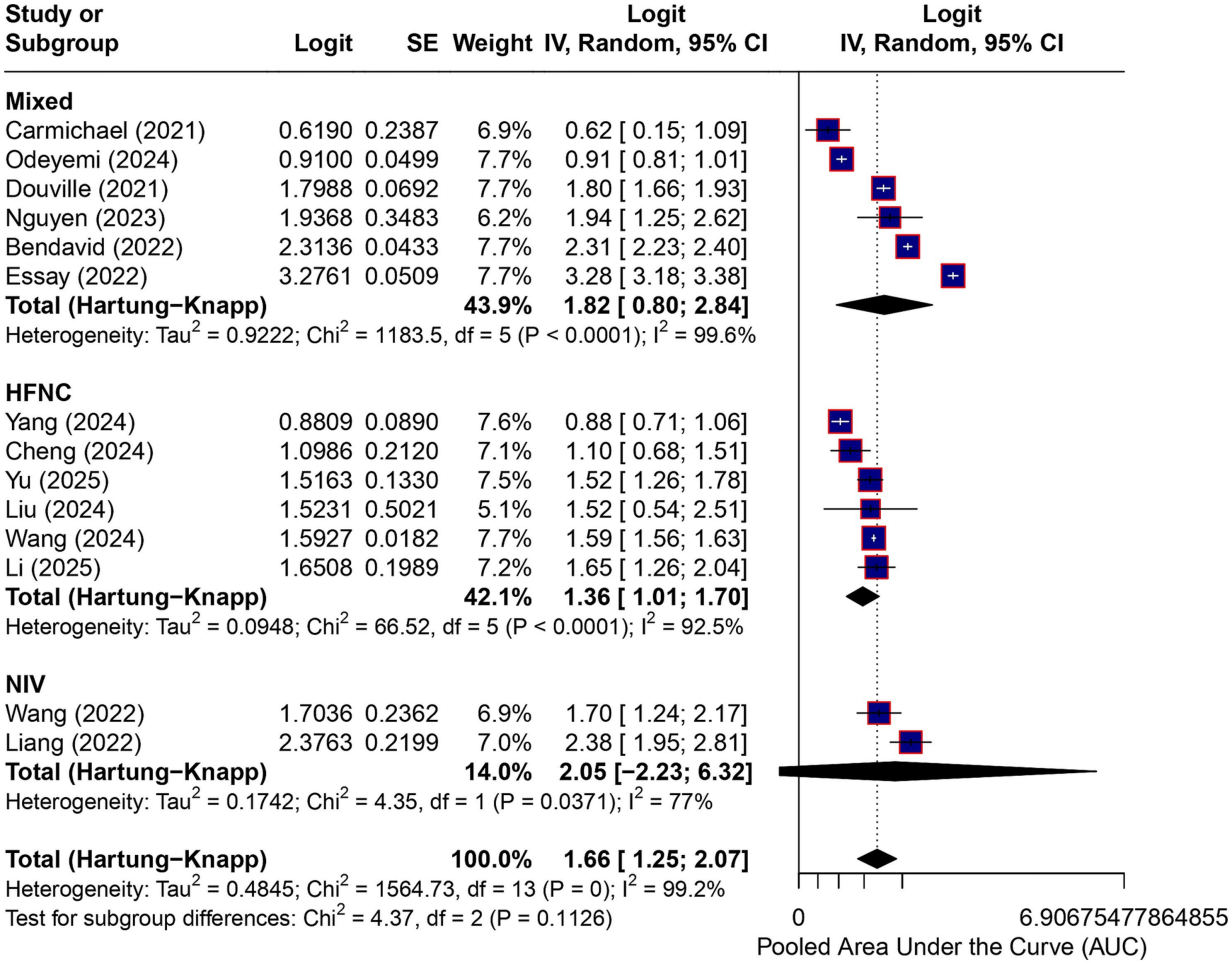
Subgroup analysis by type of noninvasive respiratory support. Subgroup analysis of discriminative performance according to type of noninvasive respiratory support (noninvasive ventilation, high-flow nasal cannula, or mixed cohorts). Effect estimates are displayed on the logit (AUC) scale and were back-transformed to AUC values for interpretation. Pooled estimates were calculated using random-effects models with restricted maximum likelihood estimation and the Hartung–Knapp adjustment. Heterogeneity remained substantial across all subgroups, with wide and overlapping prediction intervals. No statistically significant differences between subgroups were observed (*p* = 0.11). These subgroup analyses are exploratory and hypothesis-generating only and do not support inferences regarding consistent model performance across different noninvasive respiratory support modalities.

When stratified by type of noninvasive respiratory support using logit-transformed AUCs and the Hartung–Knapp adjustment, pooled AUC estimates were 0.88 for NIV-based models, 0.80 (95% CI, 0.74–0.85) for HFNC-based models, and 0.86 (95% CI, 0.69–0.94) for mixed cohorts. Substantial heterogeneity persisted across all strata, with wide and overlapping prediction intervals. No statistically significant differences were detected between subgroups (*p* = 0.11). Subgroup estimates overlapped substantially, and residual heterogeneity remained high; therefore, these analyses should be interpreted as exploratory and hypothesis-generating only and do not support inferences regarding consistent model performance across different noninvasive respiratory support modalities (Figure 4).

### 3.7 Etiology of acute respiratory failure

**Figure 5:**
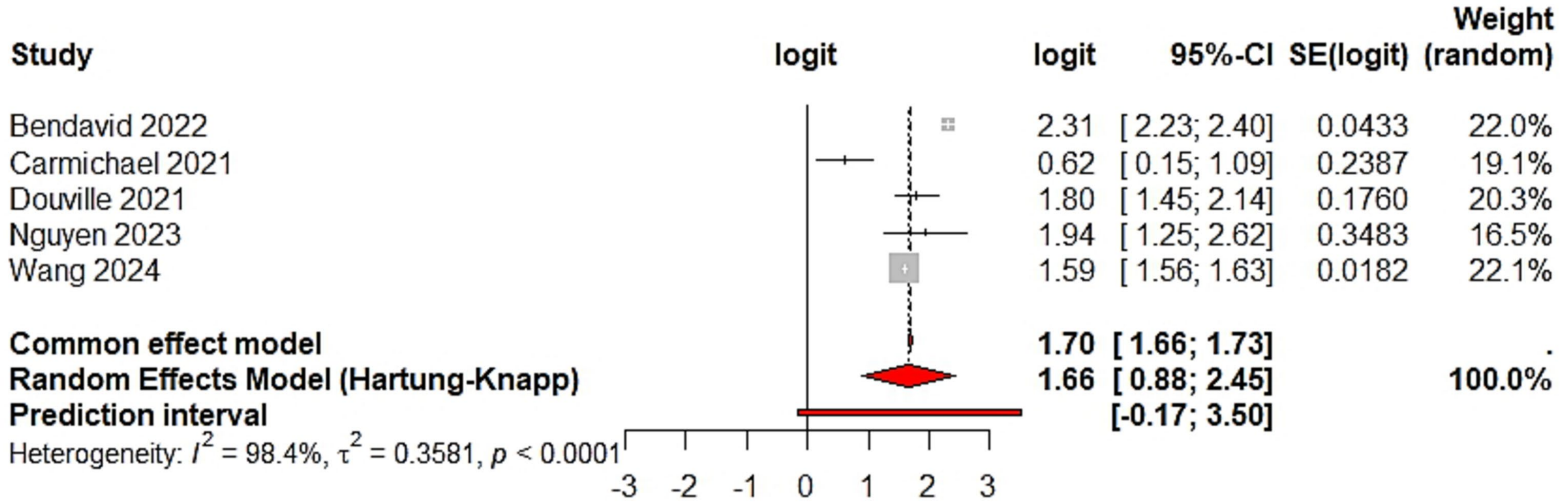
Etiology of acute respiratory failure. Forest plot illustrating the discriminative performance of machine learning–based models in a subgroup of studies specifically focusing on patients with COVID-19-related acute respiratory failure (*n* = 5). Effect estimates are displayed on the logit (AUC) scale and were back-transformed to AUC values for clinical interpretation. Summary estimates were derived from a random-effects model using restricted maximum likelihood (REML) estimation with the Hartung–Knapp adjustment for confidence intervals. Despite the shared etiology, statistical heterogeneity remained extreme (*I*^2^ = 98.4%), as reflected by the wide prediction intervals. This analysis indicates that etiology-specific stratification alone does not mitigate the substantial inconsistency in model performance, highlighting the impact of other methodological variations such as timing and outcome definitions.

To address the potential impact of patient etiology on model performance, a quantitative subgroup analysis was performed for studies specifically focusing on COVID-19-related acute respiratory failure (n = 5). The pooled AUC for the COVID-19 subgroup was 0.84 (95% CI: 0.71–0.90) under the random-effects model. Despite the shared etiology, extreme statistical heterogeneity was observed within this group (*I*^2^ = 98.4%, *p* < 0.001) (Figure 5). A similar quantitative subgroup analysis could not be conducted for the remaining studies, as they predominantly involved heterogeneous populations categorized as “General ARF.” These cohorts encompassed a wide variety of underlying conditions (e.g., pneumonia, post-operative respiratory failure), making further etiology-specific stratification statistically unfeasible and clinically inconsistent.

### 3.8 Sensitivity analyses

Sensitivity analyses excluding the largest study and studies reporting extreme AUC values yielded pooled estimates that were directionally consistent with the primary analysis, although heterogeneity remained high. Leave-one-out analyses demonstrated that no single study fully accounted for the observed variability in effect estimates, confirming that heterogeneity was distributed across studies rather than driven by a single influential dataset. These findings further support a descriptive rather than inferential interpretation of pooled performance metrics (Supplementary materials 2, 3).

### 3.9 Risk of bias assessment

**Figure 6:**
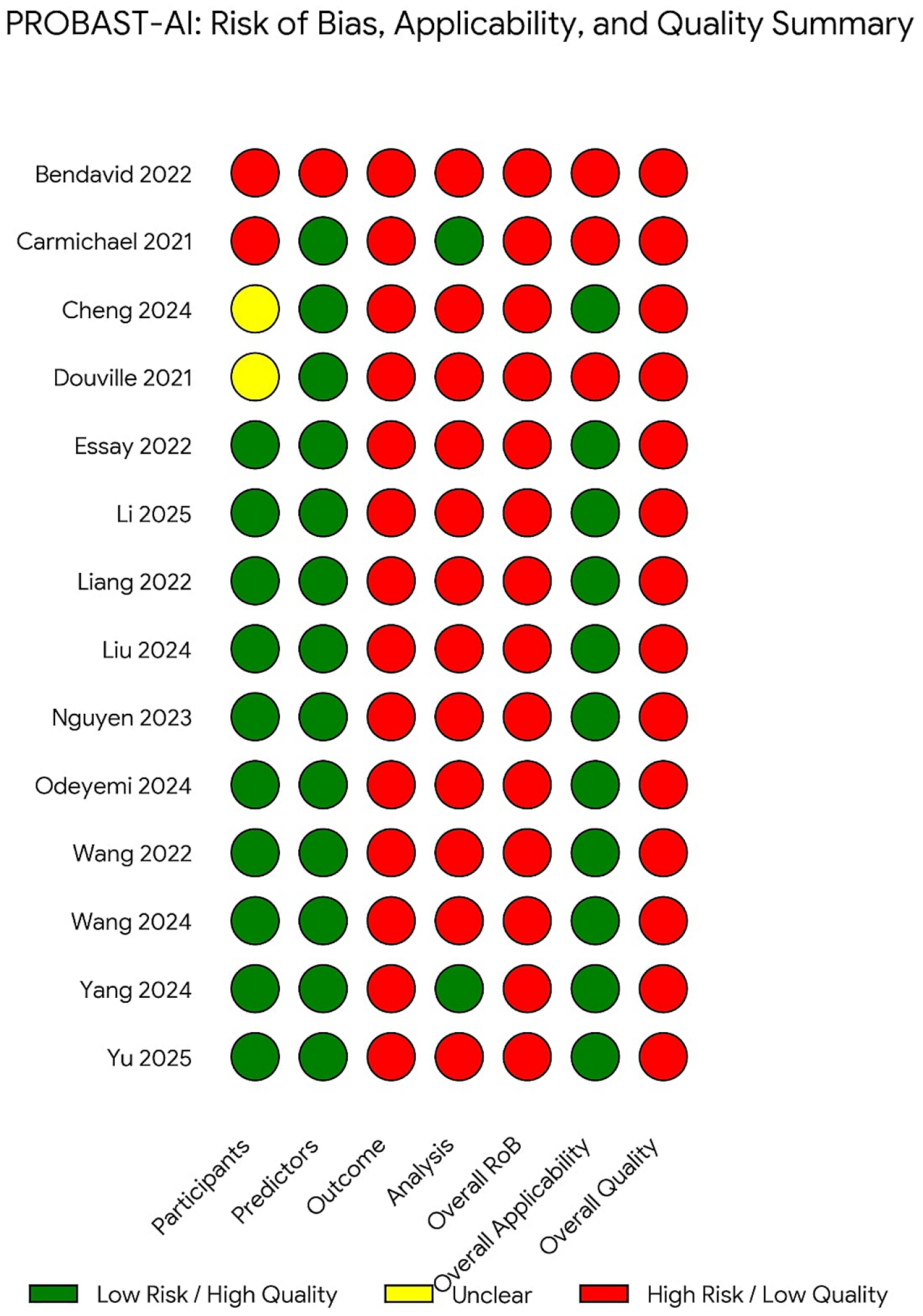
Risk of bias assessment using PROBAST-AI. Summary of risk of bias and applicability assessments for included studies using the PROBAST-AI tool. All studies were judged to be at high risk of bias, primarily due to limitations in the outcome and analysis domains, including heterogeneous outcome definitions, reliance on clinician-driven intubation decisions, incomplete reporting of model development procedures, and limited safeguards against overfitting and data leakage.

Using the PROBAST-AI tool, all included studies were judged to be at high risk of bias. The most frequently affected domains were Outcome and Analysis. Specifically, based on our PROBAST-AI extraction, 78.5% (11/14) of the included studies utilized subjective, clinician-dependent outcomes (e.g., intubation decisions) rather than standardized physiological criteria. In the Analysis domain, 85.7% (12/14) of the models lacked calibration assessments or clinical utility analysis (such as Decision Curve Analysis). Additionally, 71.4% (10/14) of the studies exhibited inadequate handling of missing data or lacked robust external validation. A summary of risk-of-bias assessments is presented in Figure 6 and detailed results in Supplementary File.

### 3.10 Certainty of evidence

**Figure 7:**
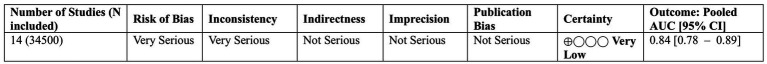
Certainty of evidence assessment (GRADE). GRADE evidence profile summarizing the certainty of evidence for the primary outcome (pooled AUC). The certainty of evidence was rated as very low, primarily due to uniformly high risk of bias and extreme inconsistency across studies. This rating indicates substantial uncertainty regarding the true predictive performance of machine learning–based models for noninvasive respiratory support failure.

According to the GRADE framework adapted for prognostic research, the overall certainty of evidence for the primary outcome was rated as very low. This rating was primarily driven by the uniformly high risk of bias and extreme inconsistency across studies, despite the relatively narrow confidence interval around the pooled AUC estimate. The GRADE evidence profile is shown in Figure 7.

## 4 Discussion

In this systematic review and meta-analysis, we evaluated the discriminative performance of machine learning–based prediction models for failure of noninvasive respiratory support in adults with acute respiratory failure. Across 14 cohort studies, the pooled AUC was 0.84 (95% CI, 0.78–0.89), indicating moderate discrimination at face value, rather than a robust or definitive level of performance. However, this summary estimate was accompanied by extreme between-study heterogeneity, uniformly high risk of bias as assessed by PROBAST-AI, wide prediction intervals, and very low certainty of evidence according to the GRADE framework. Collectively, these factors substantially limit the interpretability and applicability of pooled performance estimates, despite the use of robust statistical adjustments such as logit transformation and the Hartung–Knapp method. Furthermore, a clear distinction must be established between traditional diagnostic/prognostic tasks and treatment escalation prediction tasks. The models identified in this systematic review predominantly function as escalation-support tools. Unlike prognostic models that predict an independent biological outcome, these ML tools model the clinical decision-making process for intubation. This distinction is vital because, in the absence of standardized protocols, these predictions inherently reflect clinician behavior and institutional escalation practices rather than purely physiological transitions.

Although several machine learning models reported high discrimination within individual studies (16–18), the wide dispersion of AUC values across clinical settings highlights the absence of a consistent or reproducible level of predictive performance. Differences in patient populations, etiologies of acute respiratory failure, definitions of noninvasive respiratory support failure, timing of outcome assessment, model architectures, and validation strategies likely contributed to the observed heterogeneity. Importantly, these factors represent deep systematic differences rather than mere random variation. Because failure definitions are highly decision-dependent and local escalation rules vary widely, a machine learning model trained in one specific clinical context may exhibit poor transportability to another setting. Accordingly, the pooled AUC should be interpreted strictly as a descriptive summary of reported discrimination rather than as an estimate of expected accuracy in future clinical practice, in line with recommendations for prognostic model evaluation.

Subgroup analyses stratified by validation strategy (19–21) and by type of noninvasive respiratory support (22–24) did not demonstrate statistically significant differences in discriminative performance. Although numerical differences were observed, subgroup estimates overlapped substantially and residual heterogeneity remained high. These findings do not support inferences regarding model robustness, generalizability, or stability of performance across validation strategies or respiratory support modalities and should be interpreted as exploratory only, consistent with methodological guidance for subgroup analyses in meta-analyses.

The methodological quality of the included studies further constrains interpretation. All studies were judged to be at high risk of bias according to PROBAST-AI, particularly within the Outcome and Analysis domains (25, 26). Failure of noninvasive respiratory support was commonly defined by clinician-driven decisions to intubate, often without standardized criteria, introducing substantial potential for outcome misclassification and outcome bias. Consequently, these models may inadvertently learn to predict local physician behavior and practice patterns rather than true patient physiological deterioration. In addition, many studies lacked transparent reporting of data preprocessing, handling of missing data, prevention of data leakage, or safeguards against overfitting, as recommended by TRIPOD-AI (12, 27, 28). Furthermore, the overwhelming majority of studies focused exclusively on discrimination metrics (AUC), critically omitting calibration assessments (e.g., calibration plots) and clinical utility analyses (e.g., decision curve analysis). These limitations are likely to inflate apparent model performance and reduce reproducibility when models are applied outside the original study setting.

The certainty of evidence for the primary outcome was rated as very low using the GRADE framework, driven primarily by extreme inconsistency and high risk of bias. In prognostic research, very low certainty indicates that the true predictive performance of these models may be substantially different from the observed estimates. Consequently, current evidence does not support the routine clinical use of machine learning–based models to guide escalation decisions in acute respiratory failure.

This review has important limitations. First, the literature search did not include EMBASE or CENTRAL, and inclusion was restricted to English-language publications, which may have resulted in incomplete study capture. Additionally, while the absence of specialized registries or gray literature searches could miss unpublished models and increase publication bias risk, this restriction was intentionally applied to maintain a rigorous baseline of peer-reviewed quality amidst an already highly heterogeneous body of evidence. Second, extraction of performance metrics from the best-performing model within each study may have introduced optimism bias, despite prioritization of external validation results when available. Third, the quantitative synthesis focused exclusively on discrimination metrics; calibration, clinical utility, and impact on decision-making could not be assessed due to inconsistent reporting. Finally, pooling AUC values, even using robust statistical methods, remains challenging in the presence of extreme heterogeneity and should be interpreted cautiously, as emphasized in methodological guidance for prognostic meta-analyses.

Despite these limitations, the findings highlight clear priorities for future research rather than immediate clinical applicability. Prospective, multicenter studies with standardized definitions of noninvasive respiratory support failure, transparent reporting aligned with TRIPOD-AI, rigorous external validation, and systematic evaluation of calibration and clinical utility are urgently needed. Without such methodological advances, the clinical value of artificial intelligence–based prediction models in acute respiratory failure will remain uncertain.

To bridge the gap between research and clinical practice, we propose the following Minimum Requirements for Clinical Implementation of ML models in ARF management:

- Mandatory External Validation: Models must demonstrate performance stability across diverse, multi-center cohorts to ensure geographic transportability.
- Comprehensive Calibration Reporting: Beyond AUC, researchers should provide calibration slopes and Brier scores to confirm that predicted probabilities align with observed risks.
- Standardized Outcome Definitions: Future studies should use pre-specified physiological triggers for ‘failure’ to reduce the influence of subjective clinician decisions.
- Clinical Utility Assessment: Implementation of Decision Curve Analysis (DCA) to quantify the net benefit of model-guided decisions over standard clinical judgment.
- Methodological Transparency: Strict adherence to TRIPOD-AI reporting guidelines and the sharing of open-source code to allow for independent replication.

## 5 Conclusion

Although machine learning–based prediction models appear to show moderate discriminative performance at face value for predicting failure of noninvasive respiratory support in acute respiratory failure, the current body of evidence is characterized by extreme heterogeneity, high risk of bias as assessed by PROBAST-AI, and very low certainty according to the GRADE framework. These methodological limitations substantially restrict the interpretability and reliability of pooled performance estimates and preclude clinical implementation at present. Future research should prioritize methodologically rigorous, prospectively designed, and externally validated studies, with transparent reporting aligned with TRIPOD-AI, incorporating calibration assessments and the open sharing of algorithms, before any consideration of clinical use can be justified.

## Data Availability

The original contributions presented in the study are included in the article/Supplementary material, further inquiries can be directed to the corresponding author.

## Footnotes

1
https://www.rayyan.ai/

## Supplementary material

Supplementary Table S1 PRISMA 2020 checklist.

Supplementary Table S2 Search strategy.

Supplementary Table S3 PROBAST AI examination results.

Supplementary Figure S1 Leave-one-out sensitivity analysis for pooled AUC.

Supplementary Figure S2 Subgroup analysis based on validation type (excluding the essay study).
