# Supplementary material for: Machine learning-based prediction models for noninvasive respiratory support failure in acute respiratory failure: a systematic review and meta-analysis
Source: Front Med (Lausanne). 2026 Apr 10;13:1775670. doi: 10.3389/fmed.2026.1775670 (PMC13107651; doi:10.3389/fmed.2026.1775670)
Supplement: Supplementary Table S2 — Search strategy. [file Table_2.docx]

| **Database** | **Search string used** | **Records retrieved** |
| --- | --- | --- |
| **PubMed (MEDLINE)** | (“acute respiratory failure” OR “respiratory failure” OR AHRF) AND (“machine learning” OR “artificial intelligence” OR “deep learning” OR “neural network” OR “prediction model” OR “risk prediction”) | 482 |
| **Scopus (Elsevier)** | TITLE-ABS-KEY((“acute respiratory failure” OR “respiratory failure” OR AHRF) AND (“machine learning” OR “artificial intelligence” OR “deep learning” OR “neural network” OR “prediction model” OR “risk prediction”)) | 1,197 |
| **Web of Science (Core Collection)** | TS=((“acute respiratory failure” OR “respiratory failure” OR AHRF) AND (“machine learning” OR “artificial intelligence” OR “deep learning” OR “neural network” OR “prediction model” OR “risk prediction”)) | 572 |
