# Supplementary figures and images for: Machine learning-based prediction models for noninvasive respiratory support failure in acute respiratory failure: a systematic review and meta-analysis
Source: Front Med (Lausanne). 2026 Apr 10;13:1775670. doi: 10.3389/fmed.2026.1775670 (PMC13107651; doi:10.3389/fmed.2026.1775670)

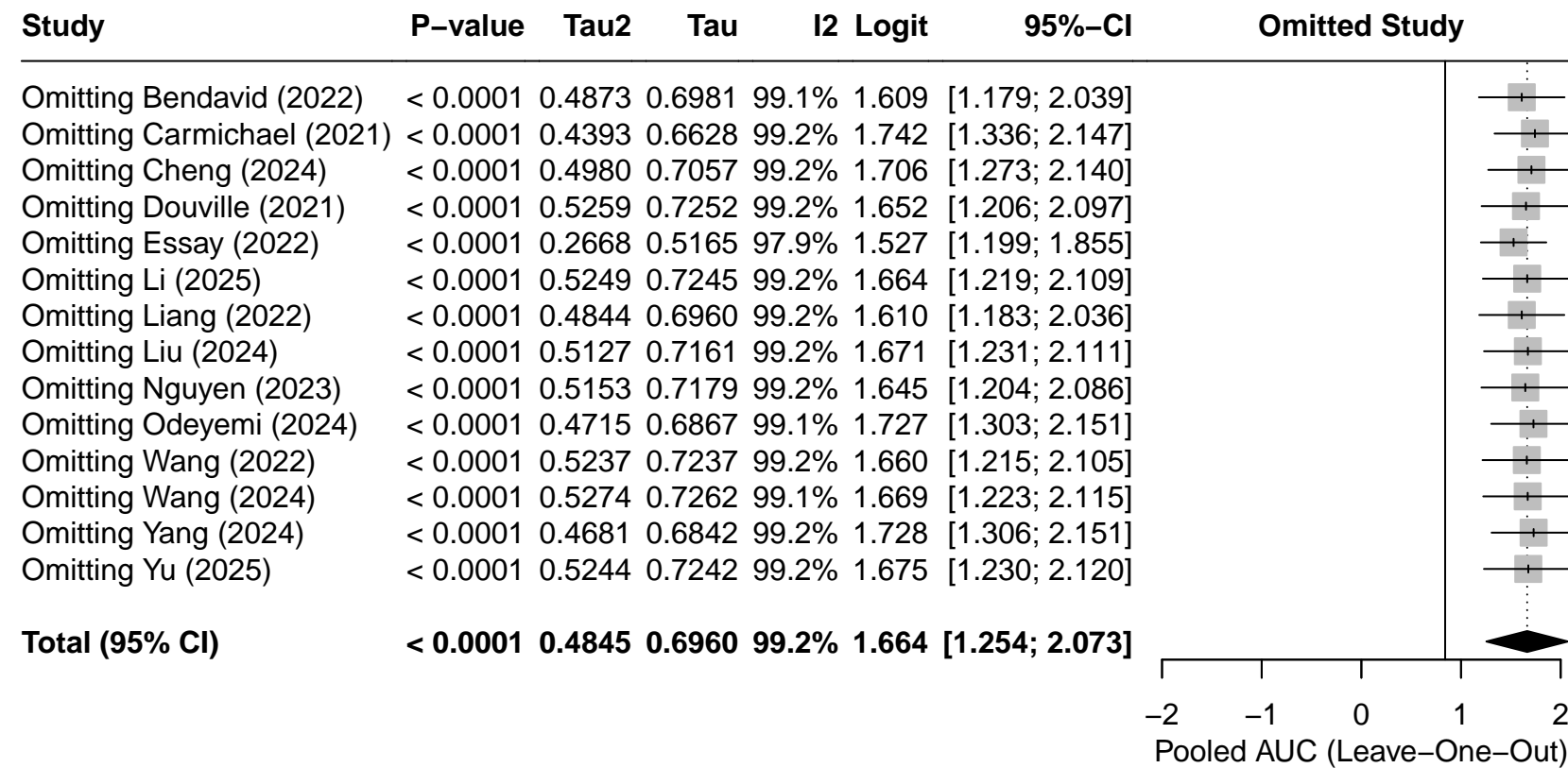

Supplement: Supplementary Figure S1 — Leave-one-out sensitivity analysis for pooled AUC. [file Data_Sheet_1.pdf]
