# Supplementary material for: Machine learning-based prediction models for noninvasive respiratory support failure in acute respiratory failure: a systematic review and meta-analysis
Source: Front Med (Lausanne). 2026 Apr 10;13:1775670. doi: 10.3389/fmed.2026.1775670 (PMC13107651; doi:10.3389/fmed.2026.1775670)
Supplement: Supplementary Figure S2 — Subgroup analysis based on validation type (excluding the essay study). [file Data_Sheet_2.pdf]

Study	Validation Type
**External**	
Carmichael (2021)	External
Cheng (2024)	External
Yu (2025)	External
Liu (2024)	External
Wang (2022)	External
Bendavid (2022)	External
**Pooled Estimate (Without Essay)**	
Heterogeneity: $I^2 = 95.4\%$, $\tau^2 = 0.3257$, $p < 0.0001$	
**Internal**	
Yang (2024)	Internal
Odeyemi (2024)	Internal
Wang (2024)	Internal
Li (2025)	Internal
Douville (2021)	Internal
Nguyen (2023)	Internal
Liang (2022)	Internal
**Pooled Estimate (Without Essay)**	
Heterogeneity: $I^2 = 97.6\%$, $\tau^2 = 0.2684$, $p < 0.0001$	
**Pooled Estimate (Without Essay)**	
Heterogeneity: $I^2 = 97.9\%$, $\tau^2 = 0.2668$, $p < 0.0001$	
Test for subgroup differences: $\chi^2_1 = 0.06$, df = 1 ($p = 0.8082$)	
